# Antimalarial activity of the 80%methanol extract and solvent fractions of *Cucumis ficifolius* A. rich roots against *Plasmodium berghei* in mice

**DOI:** 10.1016/j.heliyon.2023.e13690

**Published:** 2023-02-11

**Authors:** Gizachew Kassahun Bizuneh, Getnet Tadege, Betelhem Sirak, Abyot Endale Gurmu, Betelhem Anteneh Adamu, Aschalew Mulatu Tefera, Yeniewa Kerie Anagaw

**Affiliations:** a Department of Pharmacognosy, School of Pharmacy, College of Medicine and Health Sciences, University of Gondar, P. O. Box 196, Gondar, Ethiopia; b Department of Pharmacy, College of Medicine and Health Sciences, Mizan-Tepi University, P. O. Box 260, Mizan-Aman, Ethiopia; c Department of Pharmacy, College of Medicine and Health Sciences, Arba Minch University, P. O. Box 21, Arba Minch, Ethiopia; d Department of Pharmaceutical Analysis, School of Pharmacy, College of Medicine and Health Sciences, University of Gondar, P. O. Box 196, Gondar, Ethiopia

**Keywords:** Antimalarial activity, *Plasmodium berghei*, *Cucumis ficifolius*

## Abstract

**Ethnopharmacological relevance:**

Malaria is still a known health threat, especially in parts of sub-Saharan Africa. It is one of the frequently mentioned issues with hospital admission and outpatient care in Ethiopia. *Cucumis ficifolius* A. Rich roots are historically used in Ethiopia to treat meningitis, inflammation, and malaria. However, the antimalarial activity of this plant has not been scientifically studied so far.

**Aim of the study:**

This study aimed to determine the *in vivo* antimalarial activity of 80% methanol extract and solvent fractions of the roots of *Cucumis ficifolius* against *Plasmodium berghei* infection in mice.

**Methods:**

The i*n vivo* antimalarial activity of the 80% methanol extract and solvent fractions of *Cucumis ficifolius* A. Rich was evaluated by standard chemo suppressive, curative and repository tests using *Plasmodium berghei* (ANKA strain) in Swiss albino mice at doses of 100, 200 and 400 mg/kg/day. The level of parasitemia, survival time, variation in weight, rectal temperature, and packed cell volume of mice were determined to establish the activity of the extracts.

**Result:**

The 80% methanol extract of *Cucumis ficifolius* A. Rich roots had a promising suppression of parasitemia at 400 mg/kg with a chemosuppression value of 65.21 ± 1.20%. Among the solvent fractions, the chloroform fraction showed the highest antimalarial activity in the four-day suppressive test with a chemosuppression value of 55.9 ± 0.28%, followed by the n-butanol (42.9 ± 0.24%), and aqueous (40.57 ± 0.52%) fractions at a dose of 400 mg/kg. The highest survival times were observed with crude extract (15.4 ± 0.24 days) at 400 mg/kg, and chloroform fraction (13.4 + 0.24 days), though all extracts increased survival time.

**Conclusion:**

The findings of the present study collectively indicate the root extract of *Cucumis ficifolius* has a promising antiplasmodial activity which substantiates the traditional claim of the plant.

## 1 Introduction

Malaria is an infectious disease spread by the bite of a female Anopheles mosquito infected with the parasite [1]. In 2020, an expected 241 million malaria cases and 627,000 malaria deaths were recorded from 85 countries globally, with 228,000,000 cases reported from Africa [2]. The five Plasmodium species known to cause malaria in humans are *Plasmodium falciparum, Plasmodium vivax, Plasmodium ovale, Plasmodium malariae, and Plasmodium knowlesi* [3]. Among the *Plasmodium species, P. falciparum and P. vivax* are the most virulent agents of human malaria, causing significant global morbidity and mortality [4]. Controlling malaria parasites is getting increasingly challenging due to an increase in incidences of resistance to the bulk of currently available treatments [5]. Despite decades of intensive research and development efforts, there is currently no commercially available malaria vaccine. As a result, chemotherapeutic agents remain in great demand for the entire treatment of malaria, and the rising rate of resistance necessitates the development of new antimalarial medications [6].

Various plants have long been used to prevent, treat, and/or ease the symptoms of malaria, in addition to clinically approved drugs [7]. More than 80% of Africans, especially Ethiopians relied on medicinal plants to fulfill their basic healthcare needs [8]. Malaria has contaminated the bulk of Ethiopian's land, and 68% of the population lives in such places [9]. Due to cultural acceptance, low cost, and availability, medicinal plants are the primary source of drugs for a variety of health conditions in Ethiopian traditional medicine, including malaria [8]. More than 200 medicinal plants are utilized to treat malaria in Ethiopia [10]. However, nothing is known about the therapeutic plant's purity, consistency, and usefulness [8].

*Cucumis ficifolius* A. Rich is a prostrate perennial herb with 1 m long stems [11]. In Ethiopia, herbal preparations containing *C. ficifolius* A. Rich have been used for the treatment of many ailments including malaria [12,13]. The plant is also used to treat bloody diarrhea, wound, inflammation, nociceptive pain, and to expel ear-mites [14]. In addition, the roots are used for the treatment of rabies, meningitis, stomachache, and epistaxis [15]. The crude extracts from fruit, leaf and seed parts of *C. ficifolius* were also tested and displayed antimicrobial activities. From the root extracts of *C. ficifolius*, three anti-microbial compounds named α-spinasterol, cucurbitacin B, and cucurbitacin D were isolated [16]. The crude and solvent fraction of *C. ficifolius* showed pronounced analgesic and anti-inflammatory activities [14]. Previous phytochemical screening on the methanol extract of *C. ficifolius* has revealed the presence of phenols, tannins, saponins, terpenoids, and flavonoids [11]. Despite the traditional use of the roots of *C. ficifolius* to treat malaria, the antimalarial activity of this plant has not been scientifically studied so far. Therefore, this work dealt with *in vivo* anti-malarial activities of various extracts of *C. ficifolius* root in mice.

## 2 Materials and methods

### 2.1 Experimental plant

The fresh roots of *C. ficifolius* were collected in March 2022 from the vicinity of Gondar town, Northern Ethiopia where they used as a traditional treatment for malaria. The plant was selected on the ethnopharmacological use as an antimalarial through interviews with local communities. The fresh roots were wrapped with plastic sheets during transportation. The National Herbarium, Department of Plant Biology and Biodiversity Management, College of Natural Sciences, Addis Ababa University, confirmed and deposited voucher specimens (AE005) for future reference. After collection, the roots were properly cleansed with running tap water, sliced into smaller pieces, dried at room temperature, crushed into a coarse powder with a mortar and pestle and then stored in a glass container.

### 2.2 Experimental animals and parasites

The plant extracts were tested on either sex of healthy Swiss albino mice weighing 24–30 g and aged 6–8 weeks. The mice used in this investigation were obtained from the Addis Ababa University, School of Pharmacy, Department of Pharmacology animal house. They were kept in an air-conditioned cage at room temperature 22±3 °C, a 12-h light/dark cycle, and were allowed to acclimatize for seven days to laboratory conditions before the commencement of the experiment. The animals were fed with normal standard pellets and water *ad libitum* during the acclimatization*.* The Ethiopian Public Health Institute provided chloroquine-sensitive *P. berghei* (ANKA strain) mice, which were serially passed blood from infected animals to healthy mice once a week. The study mice were handled and cared for in accordance with the guidelines for the care and use of experimental animals [17]. Ethical approval was obtained from the Ethical Review Board of the School of Pharmacy, College of Medicine and Health Sciences, University of Gondar (ethical approval number, SOP 4/377/14).

### 2.3 Extraction and fractionation

Around 500 g of powdered plant material was extracted by maceration (100 g of dried root in 500 ml of 80% methanol) for 72 h. The extraction process was facilitated by using an orbital shaker at 120 rpm. The extract was separated from the mark using filter paper (Whatman number 1), and the residue (mark) was re-macerated for 72 h twice with fresh solvent and filtered. The filtrates were combined and then dried using a rotary evaporator (BUCHI Rotavapor TM R-300, Switzerland). A freeze dryer was used to further dry the extract. Afterward, 20 g of dried crude extract was successively fractionated using chloroform, n-butanol, and pure water. To this effect, the crude extract was suspended in water (200 ml) and then transferred to a separatory funnel. The suspension was then shaken in a separatory funnel by adding chloroform (200mlx3), and the chloroform fraction was obtained. The aqueous residue was then shaken with n-butanol (200mlx3) to obtain the n-butanol fraction. The solvents in the chloroform and n-butanol fractions were evaporated using a rotary evaporator, and aqueous residue was dried and concentrated using a lyophilizer to obtain the aqueous fraction. The calculated percent yields of the dried chloroform, n-butanol, and water fractions were 5 g (25%), 8 g (40%), and 7 g (35%). The dried crude extracts and the fractions were then transferred to separate vials and stored in a freezer (−20 °C) until use.

### 2.4 Acute oral toxicity test

According to the organization for economic cooperation and development 425, the crude extract's and its solvent fractions' acute oral toxicity was assessed [18]. The toxicity profile of the 80% methanol extract and solvent fractions were evaluated using twenty healthy female mice. All mice were fasted for 4 h before and 2 h after dosing. One mouse from each group was first administered 2 g/kg of the 80% methanol extract and fractions as a single dose via oral gavage. Then, any signs of toxicity and/or mortality were observed for 24 h with special emphasis on the first 4 h. Since no death was observed within 24 h, additional four mice were used and administered the same dose of 80% methanol extract and fractions. Then the mice were observed individually for 4 h with 30 min interval and then for 14 consecutive days once daily for the general signs and symptoms of toxicity, food and water intake, and mortality [18].

### *2.5* *In vivo* antiplasmodial activity study

#### 2.5.1 Parasite inoculation

All through the study, mice with a parasitemia level of about 30% were utilized as donors for the chloroquine-sensitive strain of *P. berghei* ANKA [19]. Decapitated donor mice were used, and blood was extracted by severing the jugular vein. Blood was then transferred onto Petri dishes that contained trisodium citrate at a concentration of 0.5%. The collected blood was diluted with 0.9% normal saline to produce 5 × 10^7^ *P. berghei*-infected red blood cells (IRBC) in 1 ml of the blood [20]. Each mouse was administered 0.2 ml of an aliquot containing 1 × 10^7^ *P. berghei*-infected erythrocytes intraperitoneally in each antimalarial model [21].

#### 2.5.2 Four-day chemosuppressive model

The aim of this test was to assess the antiplasmodial activity of test extracts during early infection. Twenty-five mice were used for each fraction during the investigation of the fractions. As negative and positive controls, respectively, Groups I and II received 0.2 ml of distilled water and 25 mg/kg/day of chloroquine. Group III, IV, and V were treated with the crude extract of *Cucumis ficifolius* at 100 (CF100 mg/kg), 200 mg/kg (CF200 mg/kg) and 400 mg/kg (CF400), respectively [22]. Similar to the crude extract, five mice per group for each fraction were randomly assigned to three treatment groups and two control groups with the first group received 0.2 ml of the vehicle (Tween 80 2% v/v in water for the chloroform and butanol fractions or distilled water for the aqueous fraction) was provided as a negative control. The positive control group was treated with the standard, CQ25. Treatment groups were given the fractions at a dose of 100 mg/kg, 200 mg/kg and 400 mg/kg dissolved in the respective vehicle. Doses were selected based on the acute toxicity results. The middle dose was one tenth of the lethal dose (∼2000 mg/kg), higher dose was twice the middle dose, and the lower dose was half of the middle dose [23,24]. Oral gavage was used for oral administration.

Oral gavage was used to administer all of the test substances. On the first day after infection, treatment began 2 h later and persisted every day until the fourth day. On the fifth day, a blood smear was made as described in section 2.5.1, and a microscope with an oil immersion objective and a 100× magnification was used to count the parasites. The parasitemia was measured by counting 5 fields on each slide [25]. The percent parasitemia and percent parasitemia suppression were calculated using the formula below:

$$\%\;Parasitemia=\frac{Number\;of\;parasitized\;red\;cell}{Total\;red\;cell}\times 100$$

$$\%\;Supression=\frac{A-B}{A}\times 100$$

where A represents mean percentage parasitemia level of the mice in the negative controls and B represents the mean parasitemia level of the mice in the treatment groups.

#### 2.5.3 Evaluation of survival time and body weight

Body weight and mean survival days were used to measure each extract's *in vivo* antiplasmodial activity. Using a precise digital weighing scale, the experimental mice's body weight was measured on day zero before infection and day four after therapy was completed. After inoculation, the death of every mouse was monitored daily from day 1 to day 28. Then, average body weight and average survival time were determined for each group [26]. using the following formulas.

$$Mean\;body\;weight=\frac{Sum\;of\;body\;weight\;of\;all\;mice\;in\;each\;group}{Total\;number\;of\;mice\;in\;that\;group}$$

$$Mean\;survival\;time\;\left(MST\right)=\frac{Sum\;of\;survival\;times\;of\;all\;mice\;in\;each\;group\;\left(days\right)}{Total\;number\;of\;mice\;in\;that\;group}$$

#### 2.5.4 Determination of rectal temperature and packed cell volume

Rectal temperature and packed cell volume were used to measure the antimalarial effects of the test extracts in mice (PCV). To determine the PCV, blood was taken from the mice's tail and poured into capillary tubes that had been heparinized and filled to 75% of their height. The capillary tubes were sealed with sealing clay at their dry end and spun for 5 min at 11,000 rpm in a hematocrit centrifuge. Digital thermometers were used to gauge the mice's rectal temperatures. The PCV and mean rectal temperature were calculated using the formula below [27].

$$Mean\;rectal\;temprature=\frac{Sum\;of\;temperature\;of\;individual\;mouse\;in\;a\;group}{Total\;number\;of\;mice\;in\;a\;group}$$

$$PCV=\frac{Volume\;of\;erythrocytes\;in\;a\;given\;volume\;of\;blood}{Total\;blood\;volume}\times 100$$

#### 2.5.5 Evaluation of established infection

Early infection studies showed the 80% methanol extract of *C. ficifolius* to have good antiplasmodial activity, so Ryley and Peters [28] method was used to evaluate the extract's therapeutic benefits. To screen the test extracts, 25 parasitized mice were randomly divided into five groups each containing five mice (72 h after inoculation). The mice were administered the test substances outlined in section 2.5.2 and continued for three days. A thin blood film was prepared (as stated in section 2.5.1) on days three to seven (daily for five days) to assess the mean % parasitemia levels. The mice's body weight, rectal temperature, and PCV were determined on the fourth day before and the eighth day following treatment. Additionally, the mice were observed for a month following infection to calculate the mean survival time [29].

#### 2.5.6 Prophylactic test (repository test)

The effectiveness of the *C. ficifolius* 80% methanol extract as a preventative strategy was assessed using Peters' approach because it had a significant curative potential [30]. Similar to the previous procedures, 25 mice were divided into 5 groups of 5, each of which contained 5 mice for each extract. From day 0 to day 3, each group received adequate treatment. All the mice were infected on the 5th day (day 4). Blood smears were made, and the parasitemia levels determined on the 8th day (day 7). The mice's body weight, temperature, and PCV were measured on the 5th day before infection and the 8th day of the experiment. The survival period of the mice was monitored for a month (30 days) post-infection, and the mean survival time was calculated.

### 2.6 Data analysis

The information was entered into a Microsoft Excel 2019 spreadsheet and then transferred to SPSS version 26 for analysis. The graph was constructed by using GraphPad Prism version 9. A mean and standard error of the mean were used to express the results (SEM). Tukey's post hoc test was performed to assess mean differences between groups, and one-way analysis of variance (ANOVA) was employed to determine statistical significance. A P value of less than 0.05 was used to determine statistical significance.

## 3 Results

### 3.1 Acute toxicity

Acute toxicity test conducted to determine the safety level of 80% methanol extract and solvent fractions showed that neither the 80% methanol extract nor the solvent fractions caused gross behavioral changes or treatment-related deaths within 24 h as well as in the following 14 days, indicating that the LD_50_ values of the extract and fractions were greater than 2000 mg/kg in mice.

### 3.2 Suppressive test

**Fig. 1:**
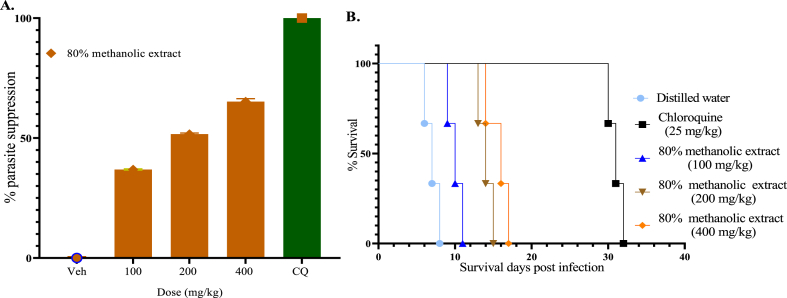
% Parasitemia suppression of the 80% methanol extract in the four-day suppression test (A), Survival days of each mouse treated with the 80% methanol extract at 100, 200, and 400 mg/kg and, chloroquine 25 mg/kg (CQ), and distilled water in the early infection (B). Values are interpreted as mean ± SEM; n = 5.

In the four-day suppressive test, the 80% methanol extract significantly reduced (p < 0.001 in all cases) parasitemia in a dose-dependent manner compared to negative control, with a percentage chemosuppression value of 36.9, 51.64 and 65.21% at various doses used in this study (100, 200, and 400 mg/kg, respectively) (Fig. 1A). Similarly, the extract at all the doses tested was significantly prolonged (p < 0.001) mean survival time of treated mice (Fig. 1B). However, the positive control had superior suppressive activity and increased survival time better than the extract.

**Fig. 2:**
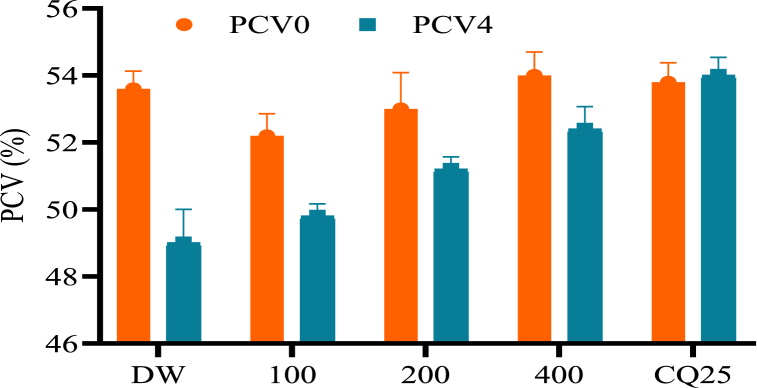
Effects of the 80% methanol extract of *Cucumis ficifolius* on packed cell volume of *Plasmodium berghei*-infected mice in the four-day suppressive test. Data expressed as mean ± SEM (*n* = 5); CQ = chloroquine; DW = distilled water; Numbers = dose in mg/kg/day; D0 = day 0; D4 = day 4.

Analysis of all parameters indicated that 80% methanol extract of *C. ficifolius* significantly prevented weight loss and rectal temperature (Table 1), and PCV reduction (Fig. 2) associated with an increase in parasitemia level. However, the 80% methanol extract exhibited lower activity (*p* < 0.001) compared to the standard drug in terms of all test parameters.

**Table 1:** The effect of 80% methanol root extract of *Cucumis ficifolius* on body weight and rectal temperature with *Plasmodium berghei* infected mice in 4-day suppressive.

Test substances (mg/kg/day)	Body weight (g)			Rectal temperature (^0^C)		
	Day 0	Day 4	% Change	Day 0	Day 4	% Change
DW	26.02 ± 0.40	22.12 ± 0.56	−17.77	36.66 ± 05	33.00 ± 0.25	−11.11
CQ 25	26.28 ± 0.59	30.50 ± 0.53	13.79^a3^	36.52 ± 0.18	36.56 ± 0.16	0.10^a3^
CF 100	25.03 ± 0.44	23.04 ± 0.25	−8.64^a2,b3^	36.26 ± 0.08	34.36 ± 0.38	−4.50^a3,b3^
CF 200	24.38 ± 0.50	22.82 ± 0.49	−6.86^a3,b3^	36.42 ± 0.13	35.24 ± 0.21	−3.18^a3,b1^
CF 400	25.36 ± 0.72	24.32 ± 0.72	−4.29^a3,b3^	36.50 ± 0.13	35.32 ± 0.09	−3.34^a3,b1^

Data are expressed as mean ± SEM; n = 5; a: compared to DW, b: compared to chloroquine, c: compared to CF 100, d: compared to CF 200, e: compared to CF 400; 1: p < 0.05, 2: p < 0.01, 3: p < 0.001; CQ: chloroquine, DW: distilled water, CF: 80% methanol extract of *C. ficifolius*.

### 3.3 Curative test

**Fig. 3:**
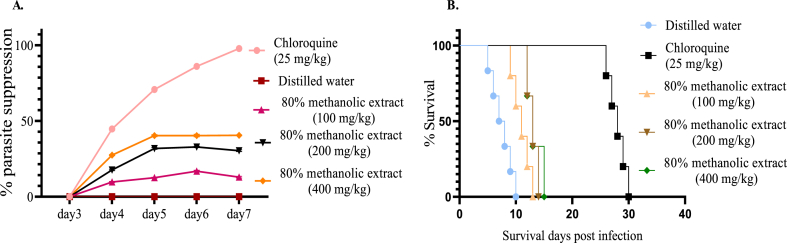
% Parasitemia suppression of the 80% methanol extract in established infection (A). Survival days of each mouse administered with methanol extract in Rane's test at a dose of 100, 200, and 400 mg/kg and, chloroquine 25 mg/kg (CQ), and distilled water (B) Values are interpreted as mean ± SEM; n = 5.

Rane's test evaluates the curative potential of the extracts on established infections [31]. There was significant parasitemia suppression in all test doses used during the course of treatment suggesting the curative potential of the 80% methanol extract of *C. ficifolius*. The 80% methanol extract at all doses exerted a significant (p < 0.001) curative effect compared to negative control group (Fig. 3A). In addition, mean survival time of extract challenged group was prolonged significantly (*p* < 0.001) at all doses as compared to negative control group (Fig. 3B). Nevertheless, the curative effect achieved with the extract was lower compared to the standard drug.

Doses at 400 mg/kg and 200 mg/kg of the extract significantly prevented body weight loss as compared to the negative control group. However, the lower dose of the extract failed to bring a statistically significant effect. Mice treated with positive control chloroquine did not lose weight as shown in Table 2. All doses of the extract significantly (*p* < 0.001) prevented the drop in rectal temperature compared to vehicle treated group (Table 2). Analysis of PCV indicated that all doses of the extract were able to significantly (*p* < 0.001) attenuate PCV reduction compared to negative control. Nevertheless, their effect was lower than the standard drug (Table 2).

**Table 2:** Body weight, rectal temperature and packed cell volume of *Plasmodium berghei*-infected mice treated with 80% methanol root extract of *Cucumis ficifolius* in Rane's antimalarial test.

Test substances (mg/kg/day)	Body weight (g)			Rectal temperature (°C)			Packed cell volume (%)		
	Day 3	Day7	% Change	Day 3	Day7	% Change	Day 3	Day7	% Change
2% TW80	26.82 ± 0.39	20.72 ± 0.25	−29.44	36.82 ± 0.08	32.38 ± 0.36	−13.74	54.80 ± 0.37	46.80 ± 0.58	−17.17
CQ 25	26.50 ± 0.51	28.10 ± 0.64	5.65^a3^	36.88 ± 0.09	36.38 ± 0.16	−1.38^a3^	54.00 ± 0.70	53.00 ± 0.70	−1.88^a3^
CF 100	25.21 ± 1.18	22.15 ± 1.44	−14.60^b2^	36.42 ± 0.21	35.05 ± 0.30	−3.94^a3^	52.50 ± 0.64	50.00 ± 0.67	−5.86^a3^
CF 200	26.48 ± 0.82	23.64 ± 0.75	−12.21^a1,b1^	36.7 ± 0.16	35.68 ± 0.20	−2.86^a3^	53.80 ± 0.58	50.20 ± 0.33	−7.16^a3^
CF 400	26.50 ± 0.78	25.32 ± 1.14	−4.70^a3^	36.70 ± 0.15	36.22 ± 0.07	−1.32^a3^	52.60 ± 0.40	49.80 ± 0.20	−5.62^a3^

Data are expressed as mean ± SEM; n = 5; a: compared to DW, b: compared to CQ, c: compared to CF 100, d: compared to CF 200, e: compared to CF 400; 1: p < 0.05, 2: p < 0.01, 3: p < 0.001; DW: distilled water vehicle, CQ: chloroquine.

### 3.4 Prophylactic test

**Fig. 4:**
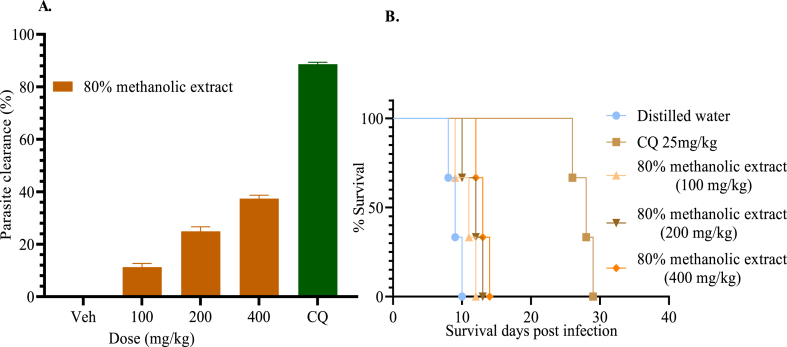
% Parasitemia suppression of the 80% methanol extract in the prophylactic test (A). Survival days of each mouse administered with 80% methanol extract (100, 200, and 400 mg/kg), chloroquine (CQ), and distilled water (B). Values are interpreted as mean ± SEM; n = 5.

The 80% methanol extract at 400 mg/kg and 200 mg/kg significantly suppressed parasitemia compared to negative control groups (Fig. 4A). All treatment groups prolonged survival time significantly compared to the negative controls, with the standard exhibiting a highly significant prolongation (*p* < 0.001) than the extract (Fig. 4B). As to the parameter of rectal temperature, all doses of the extract significantly (*p* < 0.001) prevented the reduction in rectal temperature (Table 3).

**Table 3:** Body weight, rectal temperature and packed cell volume of *Plasmodium berghei*-infected mice treated with 80% methanol root extract *Cucumis ficifolius* in prophylactic antimalarial test.

Test substances (mg/kg/day)	Body weight (g)			Rectal temperature (°C)			Packed cell volume (%)		
	Day 3	Day7	%Change	Day 3	Day7	% Change	Day 3	Day7	% Change
2% TW80	26.82 ± 0.39	22.92 ± 0.45	−17.08	36.76 ± 0.06	32.62 ± 0.22	−12.70	55.60 ± 0.50	48.00 ± 0.54	−15.90
CQ 25	26.00 ± 0.42	27.5 ± 0.43	5.44^a3^	36.62 ± 0.17	36.46 ± 0.10	−0.44^a3^	53.80 ± 0.58	53.20 ± 0.80	−1.16^a3^
CF 100	26.48 ± 0.82	24.24 ± 0.89	−9.48^b2^	36.42 ± 0.21	35.05 ± 0.30	−3.94^a3,b1^	52.50 ± 0.64	48.50 ± 0.64	−8.34
CF 200	25.21 ± 1.18	23.40 ± 1.22	−7.85^b1^	36.70 ± 0.16	35.68 ± 0.20	−2.86^a3^	53.80 ± 0.58	51.40 ± 0.74	−4.73^a2^
CF 400	29.50 ± 0.78	28.32 ± 1.14	−4.70^a1^	36.7 ± 0.15	36.22 ± 0.07	−1.32^a3^	52.00 ± 0.70	50.20 ± 0.80	−3.69^a2^

Data are expressed as mean ± SEM; n = 5; a: compared to DW, b: compared to CQ, c: compared to CF 100, d: compared to CF 200, e: compared to CF 400; 1: p < 0.05, 2: p < 0.01, 3: p < 0.001; DW: distilled water vehicle, CQ: chloroquine.

### 3.5 Antimalarial activity of solvent fractions

**Fig. 5:**
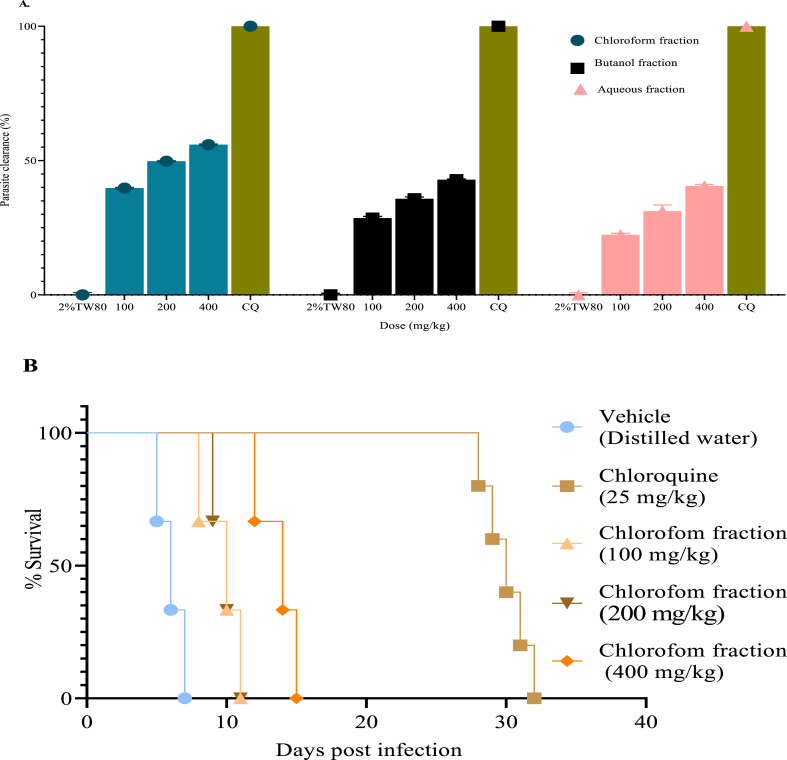
Antimalarial activity of the solvent fractions of *Cucumis ficifolius* roots against mice infected with *Plasmodium berghei* in the 4-day suppressive test. %Clearance on day-4 post-infection is calculated and survival curves of each mouse are plotted as percent survival/group for the evaluation of suppression of early-stage infection. (A) % clearance of the solvent fractions. (B) Survival plots of each mouse administered with the chloroform fraction (100, 200 and 400 mg/kg), chloroquine (CQ), and distilled water. Values are presented as mean ± SEM; n = 5.

All the fractions dose-dependently reduced parasitemia level compared to their respectivenegative control groups (Fig. 5A). Among the solvent fractions, the chloroform fraction showed the highest antimalarial activity in the four-day suppressive test with a chemosuppression of 55.9% at a dose of 400 mg/kg, followed by the butanol (42.9%), and aqueous (40.57%) fractions (Fig. 5A). All the solvent fractions significantly prolonged (p < 0.001) the mean survival time of mice compared to negative control with the highest survival time recorded for mice that received the highest dose of chloroform fraction (400 mg/kg/day) (Fig. 5B). All doses of the chloroform fraction significantly prevented (p < 0.05 for lower and middle does and p < 0.01 for the higher dose) body weight reduction (Table 4). But only the larger dose of n-butanol (p < 0.05) and aqueous (p < 0.05) fractions significantly prevented the loss of body weight associated with the increased parasitemia compared to negative control. Analysis of PCV revealed that all doses of chloroform and butanol fraction produced significant protective effect. The middle and higher doses of the aqueous fraction exhibited a significant effect against PCV reduction. However, the lower dose of this fraction didn't show statistically significant different effect.

**Table 4:** Weight, rectal temperature, and packed cell volume of infected mice treated with solvent fractions of the roots of *Cucumis ficifoluis* in the 4-day suppressive test.

Groups	Weight			Rectal temperature			Packed cell volume		
	DO	D4	%Change	D0	D4	%Change	D0	D4	%Change
2%TW80	26.6 ± 0.43	21.8 ± 0.64	−17.9 ± 1.44	37.1 ± 0.15	34.5 ± 0.18	−7.04 ± 0.66	53.4 ± 1.22	45.8 ± 0.48	−14.1 ± 1.43
CQ25 mg/kg	27.1 ± 0.69	28.1 ± 0.41	3.27 ± 0.66^a3^	36.6 ± 0.27	36.7 ± 0.24	0.28 ± 0.56^a3^	53.6 ± 0.40	53.7 ± 0.48	0.27 ± 0.87^a3^
100 mg/kg TCMF	24.7 ± 0.77	21.9 ± 0.78	−11.1 ± 2.57^a1b3^	37.1 ± 0.13	34.9 ± 0.21	−5.81 ± 0.41^b3^	53.8 ± 0.82	49.2 ± 0.86	−8.58 ± 0.6^a2b3e1^
200 mg/kg TCMF	24.7 ± 0.58	22.1 ± 0.41	−10.8 ± 1.52^a1b3^	37.1 ± 0.17	35.6 ± 0.38	−3.37 ± 0.94^a1b2^	55.6 ± 0.41	52.2 ± 0.37	−6.04 ± 0.32a^3b3^
400 mg/kg TCMF	24.5 ± 0.37	22.8 ± 0.30	−7.72 ± 0.97^a2b3^	36.6 ± 0.21	35.4 ± 0.29	−3.33 ± 0.67^a2b2^	54.4 ± 0.34	51.8 ± 0.40	−4.71 ± 0.27^a3b2^
2%TW80	26.4 ± 0.58	22.6 ± 0.81	−14.4 ± 2.06	37.04 ± 0.16	34.2 ± 0.11	−7.4 ± 0.47	55.0 ± 0.46	47 ± 1.04	−14.6 ± 2.0
CQ25 mg/kg	26.3 ± 0.48	27.6 ± 0.63	5.24 ± 2.43^a3^	36.62 ± 0.28	36.6 ± 0.92	−1.2 ± 0.82^a3^	53.8 ± 0.73	54.5 ± 0.70	1.35 ± 0.97^a3^
100 mg/kg BF	25.3 ± 0.32	22.4 ± 0.75	−11.6 ± 2.14^b3^	37.3 ± 0.16	35.2 ± 0.11	−5.3 ± 0.21^b3^	53.9 ± 0.67	49.7 ± 0.25	−7.82 ± 0.82^a2b3^
200 mg/kg BF	25.1 ± 0.61	23.0 ± 0.90	−8.19 ± 1.52^b3^	36.68 ± 0.18	35.5 ± 0.83	−3.6 ± 0.35^a3b1^	54.3 ± 0.49	50.7 ± 0.58	−6.81 ± 0.69^a3b3^
400 mg/kg BF	26.5 ± 0.46	25.1 ± 0.45	−5.27 ± 0.58^a2b2^	37.0 ± 0.19	35.6 ± 0.12	−3.6 ± 0.62^a3b1^	53.9 ± 0.92	50.4 ± 0.50	−6.30 ± 0.79^a3b2^
DW	26.8 ± 0.35	22.4 ± 0.74	−16.5 ± 1.88	37.14 ± 0.17	34.8 ± 0.21	−6.29 ± 0.45	52.4 ± 1.96	47.6 ± 1.28	−8.94 ± 1.97
CQ25 mg/kg	26.9 ± 0.61	27.1 ± 1.05	0.47 ± 2.21	36.6 ± 0.26	36.5 ± 0.24	−0.04 ± 0.74	50.2 ± 1.52	51.0 ± 1.44	1.63 ± 0.40^a3^
100 mg/kg AF	24.72 ± 0.77	22.1 ± 0.82	−10.2 ± 2.22^b2^	37.2 ± 0.16	35.5 ± 0.31	−4.77 ± 0.60^b2^	51.6 ± 1.64	49.2 ± 1.52	−4.68 ± 1.13^b1^
200 mg/kg AF	24.6 ± 0.51	22.9 ± 0.47	−6.95 ± 0.80^b1^	37.0 ± 0.18	35.7 ± 0.37	−3.61 ± 0.88^b1^	56.1 ± 0.67	54.2 ± 1.06	−3.37 ± 1.03^a1^
400 mg/kg AF	24.0 ± 0.88	23.1 ± 0.78	−4.03 ± 0.61^a2^	37.2 ± 0.13	36.3 ± 0.37	−2.47 ± 0.76^a2^	55.5 ± 0.59	54.6 ± 0.72	−1.57 ± 0.91^a2^

Data are expressed as mean ± SEM; n = 5; TCMF, chloroform fraction; BF, butanol fraction; AF, aqueous fraction; a, compared to negative control; b, to CQ25 mg/kg; c, to 100 mg/kg; d, to 200 mg/kg; e, to 400 mg/kg; 1, p < 0.05; 2, p < 0.01; 3, p < 0.001; 2% TW80, 2% Tween80; CQ chloroquine, D0 pre -treatment value on day 0, D4 post-treatment value on day 4.

**Fig. 6:**
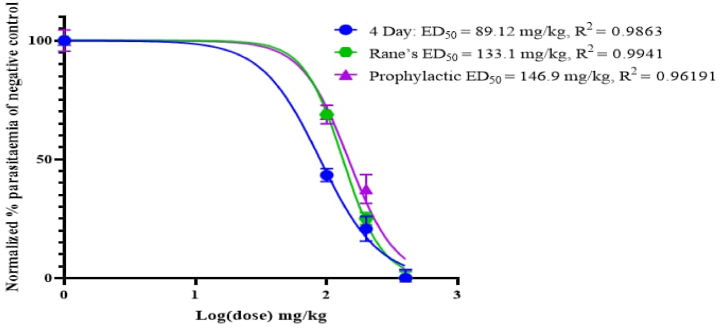
ED_50_ of 80% methanol extract of *Cucumis ficifolius* in mice infected with *Plasmodium berghei* in 4-day suppressive, Rane's, and prophylactic antimalarial tests; ED_50_ was estimated from a plot of log dose against the percentage of parasitemia of negative control group (normalized); values are presented as mean ± SEM; *n* = 5.

As shown in Fig. 6 the effective median dose (ED_50_) of 80% methanol extract of *C. ficifolius* was determined by a non-linear regression analysis from sigmoidal dose–response curves using Graph pad Prism 9.0 software. This revealed that the ED_50_s of 80% methanol extract of *C. ficifolius* are 89.12, 133.1, and 146 mg/kg in the 4-day suppressive, Rane's, and prophylactic tests, respectively.

## 4 Discussion

*Cucumis ficifolius* is traditionally used to treat malaria. However, there is no evidence that supports the long-term use and safety of this medicinal plant. The acute toxicity test result indicated that no gross behavioral changes such as impaired movement, reduced motor activity, and mortality were manifested in mice that were treated with a single dose of 2000 mg/kg until the end of 14 days. Therefore, the absence of mortality at the dose levels used in this experiment could indicate the plant was safe and this could justify the folkloric use of the plant for treating malaria. In the current investigation, the root extract of *Cucumis ficifoluis* significantly suppressed parasitemia compared to the negative control, which is similar to research on *Olea europaea* [32]. In all solvent fractions, the highest percentage of parasite suppression occurs at a maximal dose of 400 mg/kg/day. This finding implies that the active compounds of the plant that are responsible for antimalarial activity are mainly concentrated at higher doses of the extract. However, the extracts had significantly lower activity than the positive control (chloroquine). This might be due to the low concentration of active phytochemical constituent associated with the unpurified or nature of extract, low selectivity, low absorption, poor bioavailability, and other pharmacodynamics and pharmacokinetics properties of the 80% methanol extract and activity may not be detected in lower doses [19,33]. In Peter's test, the 80% methanol root extract of *Cucumis ficifolius* significantly (*p* < 0.001) suppressed parasitemia levels by 36.90, 51.64, and 65.21% at doses of 100, 200, and 400 mg/kg/day respectively. Medicinal plants are considered to have antimalarial properties when they clear at least 30% of parasites, which supported the current finding of parasite inhibition activity of the plant [34]. The 80% methanol extract of *C. ficifolius* demonstrated greater than 50% suppression at 200 mg/kg. Hence, it can be considered as a schizontocidal agent in early erythrocytic infection [35]. Moreover, the prolonged survival time of mice treated with 400 mg/kg of the 80% methanol extract is a strong indicator of the ability of the extract to reduce the overall pathogenic effect of the parasite [36].

Even though the mechanism of action of the extract was not determined, the antimalarial activities might be due to the secondary metabolites found in *C. ficifolius* root extract. According to a previous study, the methanol extract of *C. ficifolius* contains secondary metabolites including phenols, tannins, saponins, terpenoids, and flavonoids [11]. The antimalarial of phytochemicals found in 80% methanol extract may be due to inhibiting the growth, multiplication, and death of the parasite. In this regard, flavonoids exert their antiplasmodial activity by inhibiting the fatty acid biosynthesis in the parasite biochemistry [37]. They also act probably by inhibiting the influx of l-glutamine and myoinositol into infected erythrocytes during the intraerythrocytic phase of the *Plasmodium* life cycle [38]. Flavonoids exert their antimalarial action by targeting specific functional biomolecules (protein, enzymes, DNA) which are essential for parasite survival [39]. Flavonoids can make a complex with malaria protein, extracellular and other cell components, and may disturb microbial components, inactivate toxins and inhibit malarial enzymes [40,41]. Similarly, saponins exert their antimalarial effect by disorganization of membrane lipid rafts and cholesterol sequestration, and the formation of stable complexes with HMGB1 (high-mobility group box 1) proteins, including both human and *Plasmodium* [42]. The antioxidant activity of saponins [43] and phenol [26] is responsible for an antimalarial effect. Plant's antioxidant activity can inhibit the heme polymerization of malaria which is very toxic for malaria's *Plasmodium* [44]. Terpenes have been shown to have favorable antiplasmodial activity. The antimalarial mechanism behind the terpene is that it binds to the heme part of infected erythrocytes and kills the parasite just like the modern antimalarial drug artemisinin [45].

Mean survival time was another parameter used to assess the effectiveness of antimalarial medicinal plants [46]. Mice treated with 80% methanol extract of the root of *C. ficifolius* had a prolonged survival time compared to the negative control which might be due to the ability of the extract to suppress the proliferation of parasites and reduce the overall pathologic effect of the malaria parasite [47]. The prolonged survival time might be associated with the presence of flavonoids and tannins in the extract which has been proposed to act as a primary antioxidant or free radical scavengers that support the antioxidant defense system and reduce oxidative stress that is induced by the malaria parasite [48,49].

The 80% methanol extract of the root of C*. ficifolius* was fractionated by using the different polarity of solvents to further concentrate the active compound responsible for chemo suppressive effect and its activity was evaluated by Peter's test. A higher percentage of parasite suppression was attained by chloroform fraction (39.76, 49.74, and 55.90%) at doses of 100, 200, and 400 followed by butanol (28.6, 35.79, 42.9%) and aqueous fraction (22.4, 31.2, 40.57%) that is in line with the previous study [27]. Similarly, the mean survival time was more prolonged in chloroform fraction than in n-butanol and aqueous fraction. The result of the current study is in agreement with the findings reported from the previous study [49,50] where chloroform fraction of *Cucumis metuliferus* and *Osyris quadripartite* significantly suppressed parasitemia in treated mice. These results indicated the localization of a high number of active compounds in the non-polar solvent and semi-polar solvents [51].

A comparison of the antimalarial activity of the 80% methanol extract of *C. ficifolius* to that of its fractions showed that the 80% methanol extract exhibited superior antiplasmodial activity. The substantially higher efficacy of the 80% methanol extract may be due to the presence of several phytochemical components that function singly or synergistically but may be diminished or lost during fractionation. Similar results were noted; Methanol extract of *Ajuga integrifolia* produced greater chemosuppression than water, butanol, and chloroform [52]. Additionally, 80% methanol root extract of *Gardenia ternifolia* Schumach also showed higher antimalarial activity than its corresponding n-butanol, water, and chloroform fractions [21].

After observing the antimalarial effect of the root extract of the plant, further evaluation was done to confirm the curative effect of 80% methanol extract*.* In Rane's test, all doses of the 80% methanol extract significantly (*p* < 0.001) suppressed parasitemia in a dose-dependent manner. However, the curative effect achieved by the extract was lower compared to the standard drug chloroquine. The overall lower curative than the suppressive effect of the extract could be due to the short duration of action of the constituent(s) to cover the exponentially growing parasites in established infection [53]. This is in line with other studies where crude extracts had less effect on established infection than early infection [54,55].

The 80% methanol extract of *C ficifolius* produced significant (*p* < 0.001) prophylactic parasitemia suppression against *P. berghei* infection in mice compared to the negative control. In contrast to the 4-day suppressive test and the curative test, the extract had a lower percentage suppression of parasitemia in the prophylactic test. This could have resulted from fast metabolism that inactivates the active component of the extract responsible for antimalarial activity [56]. Another possibility would be that the extract might have acted through metabolic activation of the immune system and hence parasite clearance could not be total [32]. The methanol extract also extended the survival time of treated mice. The standard drug had superior activity in terms of parasite suppression and survival time.

The effect of the extract on survival time is dose-dependent which might be related to the higher percentage of parasite suppression at higher doses [53]. The 80% methanol extract shows prolonged survival time followed by chloroform fraction in a dose-dependent manner which is concordant with the previous study [57]. This might be due to the highest parasite suppression effect of the extract and the higher bioactive phytoconstituents in these extracts. The 80% methanol extract at a dose of 200 and 400 mg/kg significantly (*p* < 0.01 and *p* < 0.001) prolonged the survival time of mice as compared to the negative control. However, both the 80% methanol extract and the fraction extract exhibited significantly lower survival time than the standard drug treatment.

Weight loss, hypothermia, and anemia are common symptoms observed in *P. berghei-infected* mice [58]. Hence, effective antimalarial agents from plants prevent anemia, and weight loss and stabilize body temperature associated with the increase in parasitemia [59]. In the present study, *C. ficifolius* (in all models) and its fraction significantly prevented weight loss, PCV, and rectal temperature associated with an increase in parasitemia level compared to the negative control. However, the 80% methanol extract and solvent fractions exhibited lower activity (*p* < 0.001) compared to the standard drug chloroquine in all the test parameters.

Malaria causes anemia by inhibiting erythropoiesis and the breakup of infected RBC to release parasite merozoites [60]. Therefore, PCV is one of the parameters used to assess the antimalarial activity of medicinal plants. Plants that have secondary metabolites like flavonoids and phenols prevent malaria-induced erythrocyte hemolysis [61]. These secondary metabolites have an antioxidant and membrane-protecting effect so they have a significant PCV protective effect. Accordingly, Mice treated with 80% methanol extract (in all tests) and solvent fraction had a positive effect on preventing malaria-induced PCV reduction as compared with a negative control in a dose-dependent manner. The chloroform fraction better reverses the PCV reduction compared to other fractions which might be due to the absence of saponins in the chloroform fraction [62]. In the negative control groups, the parasitemia was increased which would destroy more red blood cells and resulted in a significant decrease in PCV. This finding is in agreement with the PCV protective effect of 80% methanol extract of *Gardenia ternifolia* Schumach and *Lobelia giberroa* Hemsl roots [21,24].

The loss of body weight in *P. berghei* infection is associated with appetite depressant action, disturbed metabolic function, and the hypoglycemic effect of the parasite [53]. All doses of 80% methanol extract significantly prevented body weight reduction compared to non-treated control groups.

A decrease in the metabolic rate of *P. berghei-infected* mice occurs before death and is accompanied by a corresponding decrease in body temperature (hypothermia) that could lead to death [63]. However, the 80% methanol extract and chloroform fraction significantly prevented the drop in rectal temperature associated with infection, and this is in line with a previous report [57]. This temperature stabilizing effect of the plant could indicate that the plant can ameliorate some pathological processes of malaria that cause a reduction in internal body temperature [63].

Other studies conducted on *C. ficifolius* revealed that 80% methanol extract root extract and solvent fractions possessed anti-nociceptive and anti-inflammatory activities [14,64]. The inflammatory condition of malaria is characterized by free radical generation and excessive release of pro-inflammatory cytokines such as tumor necrosis factor (TNF)-alpha, interleukin (IL)-12, IL-10, IL-6, and interferon (IFN)-gamma. These inflammatory cytokines up-regulate the adhesion of blood elements (Platelets and leukocytes) to the inner wall of blood vessels (endothelium). The adhering elements can set up local foci of inflammation, generating more inflammatory cytokines including inflammatory cascades initiated by HMGB1 released from the adhering activated platelets which may limit progression from uncomplicated malaria to severe complication [65,66]. As a result, the anti-inflammatory effect of *C. ficifolius* could help to reduce the overall the parasite's overall pathologic effect by inhibiting the release of cytokines and inflammatory enzymes.

The antimalarial effect of the plant could also originate from cucurbitacin, tetracyclic terpenes with steroidal structures that were isolated from plants of the family *Cucurbitaceae* having moderate antiplasmodial activity [67].

## 5 Conclusion and recommendation

The findings of this investigation supported the notion that *Cucumis ficifoluis* has strong antimalarial activity and is relatively nontoxic. With varying degrees of chemosuppression, the solvent fractions also produced antimalarial activity, with the chloroform fraction being particularly potent. To assess its long-term safety profile, sub-acute and chronic toxicity testing should be performed in the future. The *in vitro* antiplasmodial activity of the plant should be determined as the reported activity might be link to the immunomodulation effect or biotransformation. It is also crucial to understand and isolate active constituents that explain the possessed antimalarial activity in the roots of *Cucumis ficifoluis*.

## Author contribution statement

Betelhem Sirak and Gizachew Kassahun Bizuneh: Conceived and designed the experiments; Performed the experiments; Analyzed and interpreted the data; Wrote the paper. Abyot Endale Gurmu; Betelhem Anteneh Adamu; Yeniewa Kerie Anagaw: Performed the experiments; Analyzed and interpreted the data; Contributed reagents, materials, analysis tools or data. Getnet Tadege; Aschalew Mulatu Tefera: Analyzed and interpreted the data; Contributed reagents, materials, analysis tools or data; Wrote the paper.

## Funding statement

This research did not receive any specific grant from funding agencies in the public, commercial, or not-for-profit sectors.

## Data availability statement

Data will be made available on request.

## Declaration of competing interest

The authors declare no conflict of interest.
